# Reflecting realities: gauging the pulse of simulator-based training in medical minds—resonance of simulator-based ultrasound training in medical education

**DOI:** 10.1186/s12909-025-07198-4

**Published:** 2025-05-06

**Authors:** Lukas Pillong, Franziska Marietta Sprengart, Florian Recker, Maximilian Rink, Andreas Weimer, Daniel Merkel, Carlotta Ille, Holger Buggenhagen, Liv Lorenz, Anna Dionysopoulou, Roman Kloeckner, Bernhard Schick, Johanna Helfrich, Julia Weinmann-Menke, Elias Waezsada, Johannes Weimer

**Affiliations:** 1 Department for Otorhinolaryngology and Head- and Neck-Surgery, Saarland University, Kirrbergerstraße 100, 66421 Homburg, Germany; 2 Rudolf Frey Learning Clinic, University Medical Centre, Johannes Gutenberg University Mainz, 55131 Mainz, Germany; 3 Department of Obstetrics and Prenatal Medicine, University Hospital Bonn, 53127 Bonn, Germany; 4 Department of Otorhinolaryngology, Head and Neck Surgery, University Hospital Regensburg, 93053 Regensburg, Germany; 5 Center of Orthopedics, Trauma Surgery, and Spinal Cord Injury, Heidelberg University Hospital, 69120 Heidelberg, Germany; 6 BIKUS—Brandenburg Insitute for Clinical Ultrasound, Brandenburg Medical School Theodor Fontane (MHB), 16816 Neuruppin, Germany; 7 Department of Radiation Oncology and Radiotherapy, University Medical Center, Johannes Gutenberg University Mainz, 55131 Mainz, Germany; 8 Department of Gynecology and Obstetrics, University Medical Center, Johannes Gutenberg University Mainz, 55131 Mainz, Germany; 9 Institute of Interventional Radiology, Hospital Schleswig-Holstein, Campus Lübeck, University, 23562 Lübeck, Germany; 10 Department of Medicine, University Medical Center, Johannes Gutenberg University Mainz, 55131 Mainz, Germany; 11 Department of Rhythmology, Heart and Diabetes Center Bad Oeynhausen, 32545 Bad Oeynhausen, Germany

**Keywords:** Ultrasound education, Ultrasound simulators, Simulator-based training, Simulation, Medical education

## Abstract

**Background:**

Simulator-based training (SBT) transforms medical education from traditional methods to technology-driven simulations for safer, complex scenario learning. This study examines perceptions, benefits, drawbacks, and challenges of such training, focusing on ultrasound simulations among medical students and physicians.

**Methods:**

The study surveyed 343 participants: 154 third-year medical students, 97 practical-year students, and 92 physicians across various specialties. A digital questionnaire was used to analyze their views on SBT, featuring main- and sub-items evaluated through a Likert scale and dichotomous questions.

**Results:**

Widespread exposure to SBT was evident, notably in ultrasound simulator usage, where over 60% of all respondent groups reported prior experience. Significant disparities in acceptance and assessment between students and physicians were noted, particularly highlighting inconsistent integration into mandatory education and a marked deficit in physicians’ training (*p* < 0.001). All groups acknowledged the relevance of SBT for developing practical skills and patient safety. The interest in ultrasound simulator use showed variability across specialties (*p* < 0.001). While ultrasound pathology training was highly valued, doubts about simulators replacing hands-on patient experience persisted.

**Conclusions:**

Our study highlights the necessity for enhanced integration of SBT within medical curricula. It highlights the significance of adaptive teaching methodologies and singles out ultrasound simulator training as essential for practical skill development. Future research should concentrate on creating comprehensive customized teaching strategies to elevate the quality of patient care.

**Supplementary Information:**

The online version contains supplementary material available at 10.1186/s12909-025-07198-4.

## Introduction

A sound education and continuous professional development of physicians are the foundations for ensuring the quality of the healthcare system. However, technological progress, the increasing complexity of medical diagnostic and therapeutic procedures, and rising demands on medical personnel, quality standards, and patient safety [1] necessitate a secure education in dealing with these challenges. Traditional training paradigms such as “See one, do one, teach one” are no longer sufficient and are being replaced by approaches like “See one, practice many, do one” [2]. The COVID pandemic and the associated restrictions on teaching at university hospitals posed a significant obstacle to imparting practical skills in medical education. New concepts had to be developed to provide alternatives for teaching manual and valuable techniques. In this context, simulator-based training experienced a true renaissance.

Simulator-based training in the medical context has a long history [3, 4]. It has shown positive effects in numerous areas, such as training for cardiovascular and pulmonary bedside skills [5, 6], surgical abilities [7], cardiopulmonary resuscitation [8], and blood transfusion management [9]. Simulation is the artificial representation of real circumstances and conditions that promote learning in a realistic scenario through immersion, reflection, feedback, and practice while avoiding the risks associated with real experiences for patients, teachers/trainers, and students. A familiarization, especially with rare medical situations, can be trained through targeted engagement with scenarios in a safe and protected environment, thus facilitating the translation of learned skills into a clinical setting [10]. Simulator-based training offers an ideal solution as the demand grows for a medical curriculum based on cognitive, psychomotor, and affective learning domains.

Due to its versatility, wide availability, cost-effectiveness, and radiation-free nature, ultrasonography plays a crucial role in numerous medical specialties. These characteristics make it a valuable tool in medical diagnostics and patient care. Therefore, proficiency in this technique is fundamental for efficient, high-quality, and targeted patient care. In recent years, efforts have been made to integrate ultrasound courses into curricula at many universities and the clinical training of resident physicians. However, a unified teaching concept has yet to be established in Germany [11, 12]. The operator-dependency of the procedure underscores the importance of thorough training. It is essential that, alongside teaching sono-anatomical knowledge, sufficient routine in handling the ultrasound system, psychomotor skills [13], three-dimensional visualization, and spatial orientation can be trained. Therefore, a “hands-on” approach is indispensable in ultrasound education.

Positive effects have also been demonstrated for the use of simulators in ultrasound education for conducting transthoracic echocardiography [14], endobronchial ultrasound [15], FAST [16], prenatal ultrasound [17], and ultrasound-guided procedures [18]. Some course modules offered by the German Society for Ultrasound in Medicine (DEGUM) are now supported by simulators [19, 20]. However, studies on design, research questions, ultrasound applications, simulator systems used [21, 22], and specific didactic requirements for simulator-based or supported training remain highly heterogeneous. Furthermore, validation and accreditation of a particular simulator-based ultrasound training for uniform curricula still need to be established.

This study aimed to assess the experiences, perceived benefits, and challenges of simulator-based training among medical students and physicians, focusing on ultrasound training, to develop recommendations for its effective integration into medical education. Specific objectives included understanding the experiences and needs of different target groups, identifying differences in acceptance and usage between students and physicians, evaluating the effectiveness of ultrasound simulators, and exploring challenges for implementation.

## Methods

### Study design, study procedure, and overview of participants

This prospective cross-sectional survey recruited participants from university ultrasound courses and certified DEGUM courses. A total of 343 individuals took part in the study: 154 third-year medical students, 97 practical-year students, and 92 physicians from various specialties. Among the physicians, the distribution was as follows: Internal Medicine (47.8%), Surgery (19.6%), General Practice (7.6%), Anesthesiology (7.6%), Cardiology (3.3%), and smaller representations (1.1% each) from Gastroenterology, Hematology, Neurology, Oncology, Radiology, and Trauma Surgery. Emergency Medicine was represented with 2.2%. 3.3% of the physicians did not indicate their specialty.

Third-year medical students were surveyed during their semester-based echocardiography workshops. These workshops, which included six courses (each lasting approximately five hours), were adapted from previous studies [23, 24]. They focused on anatomy and physiology, device settings, transducer handling, and practical scanning to enhance imaging and address clinical challenges queries. Emphasis was placed on developing sustainable ultrasound skills, including diagnosing pathologies such as aortic aneurysms, cholecystitis, renal congestion, and pleural effusion. Simulator training was additionally offered.

Practical-year students participated in two-day workshops, which included 90-min ultrasound sessions and covered ultrasound examinations in a refresher format on the lungs, aorta, gall bladder, and kidneys. The workshop was designed based on a preliminary study to consolidate long-term skills. Simulator training also explicitly supplemented it [24].

Physician participants attended three-day DEGUM-certified basic abdominal ultrasound courses [25]. This course focused on abdominal sonography of the vascular system, liver, gallbladder/bile ducts, kidneys, and pelvic organs and explicitly integrated simulator training. In all courses, participants were offered the opportunity to use the CAE Vimedix ultrasound simulator (CAE Healthcare, Montreal, QC, Canada) as part of the practical training.

After each course, participants completed a digital questionnaire assessing their attitudes and previous experiences with simulator-based training and explicitly evaluating the ultrasound simulator. This evaluation covered various criteria, including image sharpness, handling, design, and the simulator’s effectiveness in enhancing clinical readiness, ensuring safer examinations, and improving pathology understanding. Inclusion criteria included complete participation in the course and full completion of the questionnaire.

### Questionnaire

A questionnaire was designed to comprehensively evaluate attitudes, experiences, and needs concerning simulator-based training, particularly in ultrasound education. It included demographic information (e.g., age, gender, level of training, specialty), prior experience with simulators, perceived benefits and barriers, and specific needs regarding usability and design.

The questionnaire was administered in German to ensure accessibility and understanding for all participants. It consisted of 97 items, including:

- Dichotomous questions: These questions were used to capture binary responses, such as whether participants had previous experience with simulator-based training (e.g., "Have you used a simulator before? Yes/No").
- Likert scale questions: The attitudes and perceptions of participants were evaluated using a 7-point Likert scale, ranging from "absolutely no agreement" (1) to "complete agreement" (7). We used a 7-point Likert scale to provide a higher resolution in the differentiation of responses, allowing for finer nuances in participants' opinions [26, 27]. This type of scale also includes a precise midpoint, which offers respondents an option to indicate neutrality [28]. Additionally, the increased number of response options enhances the variance in collected data, supporting robust statistical analyses [29].

#### Data collection for main aspects

The data collected through the questionnaire were organized into three main aspects:

Experiences with simulator-based training, perceived benefits and challenges, and ultrasound simulator training. The following sections describe the specific questions used to assess each element and the corresponding scales:

##### Experiences with simulator-based training

Experiences were assessed using dichotomous questions to determine prior use of simulators (e.g., "Have you used an ultrasound simulator before? Yes/No"). The focus was primarily on capturing whether participants had previously used simulator-based training and which types of simulators they had experience with. Questions related to the role of instructors, learning success, and integration of Simulator-Based Training into mandatory training were also included to provide a broader understanding of participants' experiences with different forms of simulator-based training.

##### Perceived benefits and challenges and limitations of simulator-based training

Dichotomous questions were used to identify specific reasons for or against simulator-based training in participants' institutions. For example, participants were asked questions like "What reasons do you believe support the use of simulators in your institution?" with options such as "Improvement of clinical-practical skills" (Yes/No).

Likert scale questions were also employed to measure participants' perceptions of the benefits and challenges of simulator-based training. An example includes: "To what extent do you believe simulator-based training leads to a better transfer of theory into practice?" rated on a scale from 1 ("absolutely no agreement") to 7 ("complete agreement").

##### Ultrasound simulator training

The data collection for ultrasound simulator training included both the participants' contact with ultrasound simulators and their evaluation of the effectiveness of these simulators.

Contact with Ultrasound Simulators and Demand for Training: Participants were asked whether they had previously used an ultrasound simulator and in which clinical fields they had utilized it (e.g., cardiology, abdominal, emergency medicine). The aim was to capture the specific demand for ultrasound simulator training in different medical specialties.

Evaluation of Ultrasound Simulators: The effectiveness of ultrasound simulators was assessed through Likert scale questions. Participants evaluated aspects such as image quality, ease of handling, realism, and usefulness for clinical practice preparation. An example of a Likert scale question is: "How realistic did you find the ultrasound simulator in terms of image representation?" (1 = "not realistic at all"; 7 = "very realistic").

This combined approach allowed us to comprehensively assess both the demand for ultrasound simulators and their effectiveness, providing insight into the role of ultrasound training within the broader context of simulator-based education.

The validity of the questionnaire was ensured by adhering to established guidelines in its design [30–32]. Particular emphasis was placed on creating a user-friendly layout and clearly formulated questions to ensure that all relevant aspects (such as attitudes, experiences, and needs) were adequately captured and that the consistency of responses was supported through clear instructions. To ensure secure and valid data management, 97 questions were administered using the digital survey software “SoSci” (Software Version 3.5.05; SoSci Survey GmbH, Munich, Germany). This approach provided automatic data backup and enabled direct transfer into a database, thereby preventing transcription errors and minimizing the possibility of data modification by respondents. These features were intended to ensure the reliability and validity of the entire data collection process. The reliability of the questionnaire was assessed by calculating Cronbach's Alpha for the scales used. The values obtained for the scales in this study ranged from 0.8 to 0.91, indicating good internal consistency [33].

### Ultrasound simulator

**Fig. 1:**
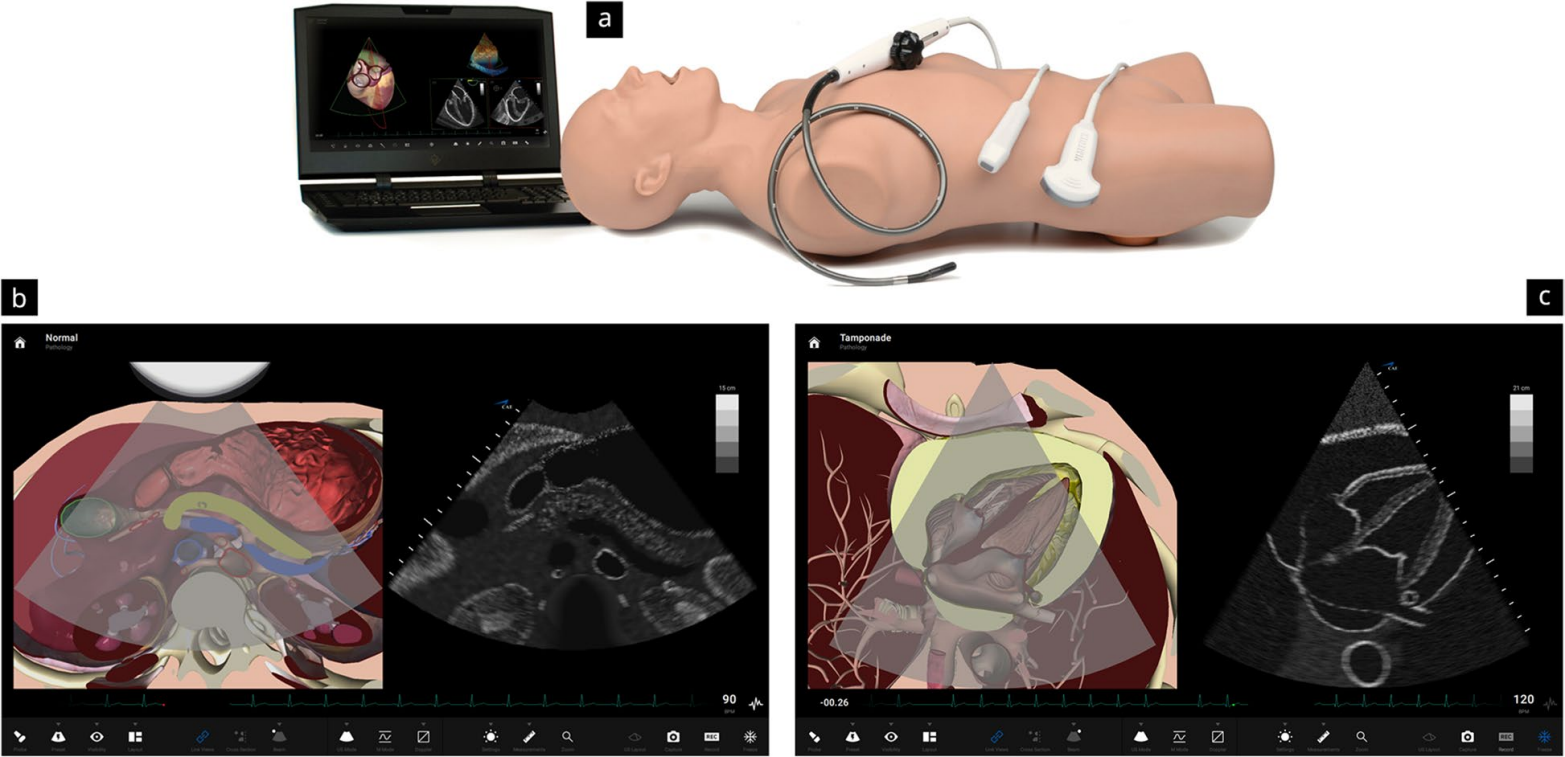
Illustration showing the CAE Vimedix ultrasound simulator used during the courses. The simulator consists of a laptop, various probes, and a dummy (**a**). Depending on the probe’s position on the dummy, the laptop screen displays a split-screen view with a schematic representation of the ultrasound image in an animation (left side) and the corresponding ultrasound image (right side). Besides normal findings (**b**), a wide selection of pathologies (**c**) can be combined for progressive training scenarios—pictures reproduced with permission from CAE Healthcare (Vimedix Ultrasound Simulator)

The ultrasound simulator (CAE Vimedix, CAE Healthcare, Montreal, QC, Canada) used as a supplement in the courses is designed to support the development of psychomotor and cognitive skills, particularly regarding the handling of ultrasound probes, image interpretation, diagnosis, and decision-making. The system consists of an ultrasound probe connected to a computer and screen and a plastic phantom that allows for an anatomical-topographical correlation depending on the probe’s position. The simulator offers animations for ultrasound training and supports in-person and remote exercises, including depicting pathologies in selected cases (Fig. 1).

### Statistical analysis

The data were acquired digitally via an online questionnaire tool and then exported to Excel for further statistical analysis. The cleaned data were imported into R Studio Version 4.0.3. To determine the main scale values, the average of the subscale values of each topic listed in Supplementary Materials Table S1 was calculated, and an overall value from the main scale values was determined. The internal consistency of the scales was confirmed using Cronbach’s Alpha. Descriptive and exploratory statistical analyses were conducted, with interval scales tested for normal distribution using Shapiro–Wilk tests. The Welch t-test with two samples was used for normally distributed scales, while the non-parametric Wilcoxon-Mann–Whitney test was employed for non-normally distributed scales. Posthoc tests were performed to compare the subscales within the different course models. Differences were analyzed using one-way ANOVA and pairwise t-tests, with corrections for multiple tests made according to Bonferroni. *P*-values below 0.05 were considered statistically significant.

## Results

### Data description

According to Cronbach’s alpha, the reliability tests show that the scales' internal consistency, which ranged from 0.8 to 0.91, did not vary considerably.

### Study population

The analysis included evaluations from 343 participants. The study population comprised 154 medical students in their third year, 97 students in their practical year (sixth year), and 92 physicians from various specialties, including internal medicine, general practice, radiology, surgery, anesthesiology, and neurology. The demographic data are presented in Table 1.

**Table 1:** Demographic data

Group	Clinical Medical Student (*n* = 154)	Internship Student (*n* = 97)	Physician (*n* = 92)	*p*-Value
	**n (%)**	**n (%)**	**n (%)**	
**Sex**				0.04
Female	89 (58)	49 (51)	43 (47)	
Male	48 (31)	43 (44)	45 (49)	
not specified	17 (11)	5 (5)	4 (4)	
**rescue personnel-preliminary education**				0.001
Yes	21 (14)	24 (25)	5 (5)	
No	133 (86)	73 (75)	87 (95)	
not specified	0 (0)	0 (0)	0 (0)	
**Period of simulator use**				0.0001
< 1 h/month	0 (0)	0 (0)	4 (4)	
> 6 h/month	11 (7)	3 (3)	2 (2)	
1 h/month	29 (19)	27 (28)	13 (14)	
2–5 h/month	17 (11)	6 (6)	1 (1)	
not specified	97 (63)	61 (63)	72 (79)	

### Study results

#### Experiences with simulator-based training

##### Exposure to simulator-based training and its application in training

Most respondents had prior experience with simulator-based training, primarily as participants (*p* = 0.0369). Only a small percentage served as instructors (*p* = 0.454), with 96% of physicians having encountered such training during their preliminary education, in contrast to significantly fewer students (*p* < 0.001). Significant differences in usage were observed in airway management, ultrasound diagnostics, gynecology, and emergency medicine. 56% of the practical year (PJ) students used simulators for airway management, compared to just 18% in the third-year students and 16% of physicians (*p* < 0.001). Similar trends were seen in emergency medicine (*p* < 0.001). For gynecology, usage was 19% among PJ students versus 1% of third-year students and 2% of physicians (*p* < 0.001). Ultrasound diagnostics were utilized by 35% of PJ students against 12% of third-year students and 8% of physicians (*p* < 0.001). Across pediatrics and other areas, the usage rate remained low for all groups, showing no significant differences (*p* = 0.1361) (see Supplementary Materials Table S2).

#### Instructors, assessment of learning success at simulators, and expansion of simulation centers

When asked about instructors for simulator-based training, 64% of PJ students and 42% of third-year students cited peers in this role, compared to only 13% of physicians (*p* < 0.001). Meanwhile, physicians were named by 62% of PJ students and 27% of both third-year students and physicians themselves (*p* < 0.001). External instructors were cited by a minority (15% of PJ students, 8% of third-year students, and 2% of physicians, *p* = 0.007). Emergency medical personnel as instructors at the simulator were reported by 25% of PJ students and 16% of third-year students but only by 9% of physicians (*p* = 0.015). The absence of instructors in this context was reported by only 1% of PJ students and 3% of physicians (*p* = 0.06619).

Regarding learning success assessment, 55% of PJ students, 32% of third-year students, and 34% of physicians reported no formal assessment (*p* = 0.0017). Practical exams as an assessment method were cited by 27% of PJ students, 37% of third-year students, and 10% of physicians (*p* < 0.001). Written tests as a means of assessing learning success were mentioned only by a few (11% of PJ students, 7% of third-year students, and 2% of physicians, *p* = 0.056).

Over the previous five years, 23% of third-year students and 32% of PJ students reported seeing an expansion of simulation centers at their institutions. In contrast, only 7% of physicians did so (*p* < 0.001) (see Supplementary Materials Table S3).

#### Integration into mandatory training and specific content delivery through

##### Simulator-based training

The assessment of the integration of simulator-based training into mandatory education varied significantly: 48% of PJ students reported such integration, compared to only 25% of third-year students and 14% of physicians (*p* < 0.001). A significant portion of physicians (34%) stated that no simulator-based training occurs in their education (*p* < 0.001). Usage in the preclinical area was low (2–10% of respondents, *p* < 0.001). In comparison, higher usage was reported in the clinical phase of medical education (56% of third-year students to 75% of PJ students, *p* < 0.001). Only 18% of physicians in postgraduate medical training report the use, against 7% of PJ students (*p* = 0.000156). Continuing education courses include simulator-based training for 27% of PJ students and 22% of physicians. Usage in training outside of.

medical studies is reported by only 2% of physicians but by 11% of third-year students and 18% of PJ students. Participants stated a demand for the use of ultrasound simulators in gynecological (7% of third-year students to 37% of PJ students, *p* < 0.001), anesthesiological, surgical, and internal medicine contents (*p* < 0.001), with a special interest in simulator-based teaching of internal medicine (54% of both third-year and PJ students) (see Supplementary Materials Table S7).

**Fig. 2:**
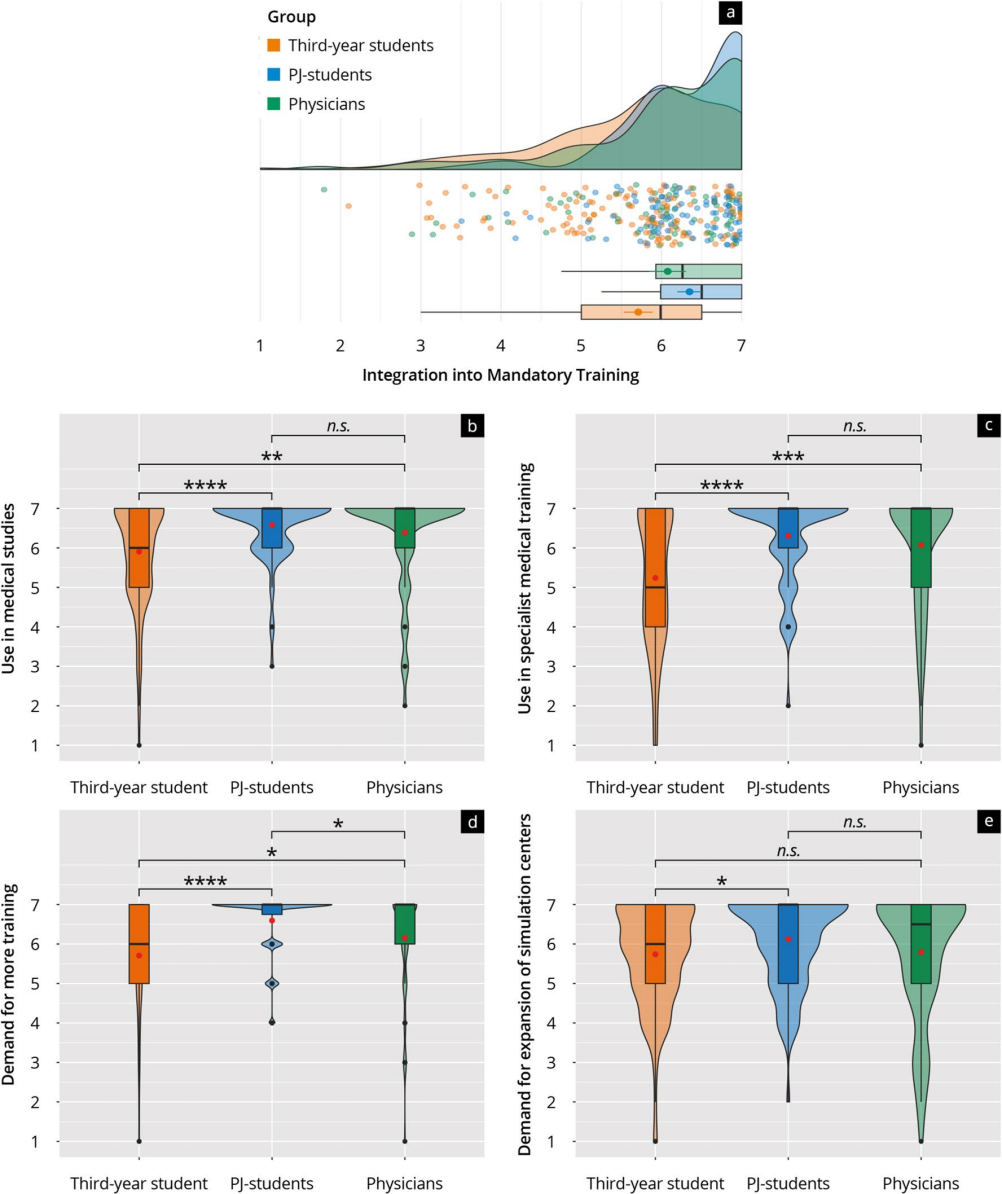
**a** Rain-plot of the mean subitem ratings for the main feature “Integration into Mandatory Training” on a Likert scale (1–7). The distributions for third-year students (orange), PJ students (blue), and physicians (green) are represented by density curves, with individual rating points displayed as a scatter below these curves. Vertical lines within each color group indicate the median and quartiles. **b**–**e** Violin plots showing the distribution of subitem ratings on a Likert scale for third-year students (orange), PJ students (blue), and physicians (green). The *p*-values as results of post-hoc tests after comparing the individual groups are indicated above the brackets, with the corresponding asterisks (* *p* < 0.05, ** *p* < 0.01, *** *p* < 0.001, **** *p* < 0.0001, n.s. = not significant)

Respondents regarded simulator-based training in medical school and specialty training positively, awarding ratings up to 6.58 (PJ students) for medical studies (Fig. 2b) and 6.07 (physicians) for postgraduate education (Fig. 2c). The demand for more training (Fig. 2d) and the expansion of simulation centers was evident, with scores of up to 6.6 for more training and 6.1 for expansion (Fig. 2e).

#### Perceived benefits and challenges of simulator-based training

The survey showed that 64% of third-year students and 84% of PJ students use simulator-based training primarily for educational purposes, unlike only 41% of physicians, of whom 47% denied this use (*p* < 0.001). Most students saw it as an opportunity to improve practical skills (56% of third-year students and 77% of PJ students versus 41% of physicians, *p* < 0.001). The didactic aspect or the innovative character of the simulation was rated mainly as being of secondary importance (*p* < 0.001), as was the argument of cooperation with external partners (*p* = 0.1253) (see Supplementary Materials Table S4).

**Fig. 3:**
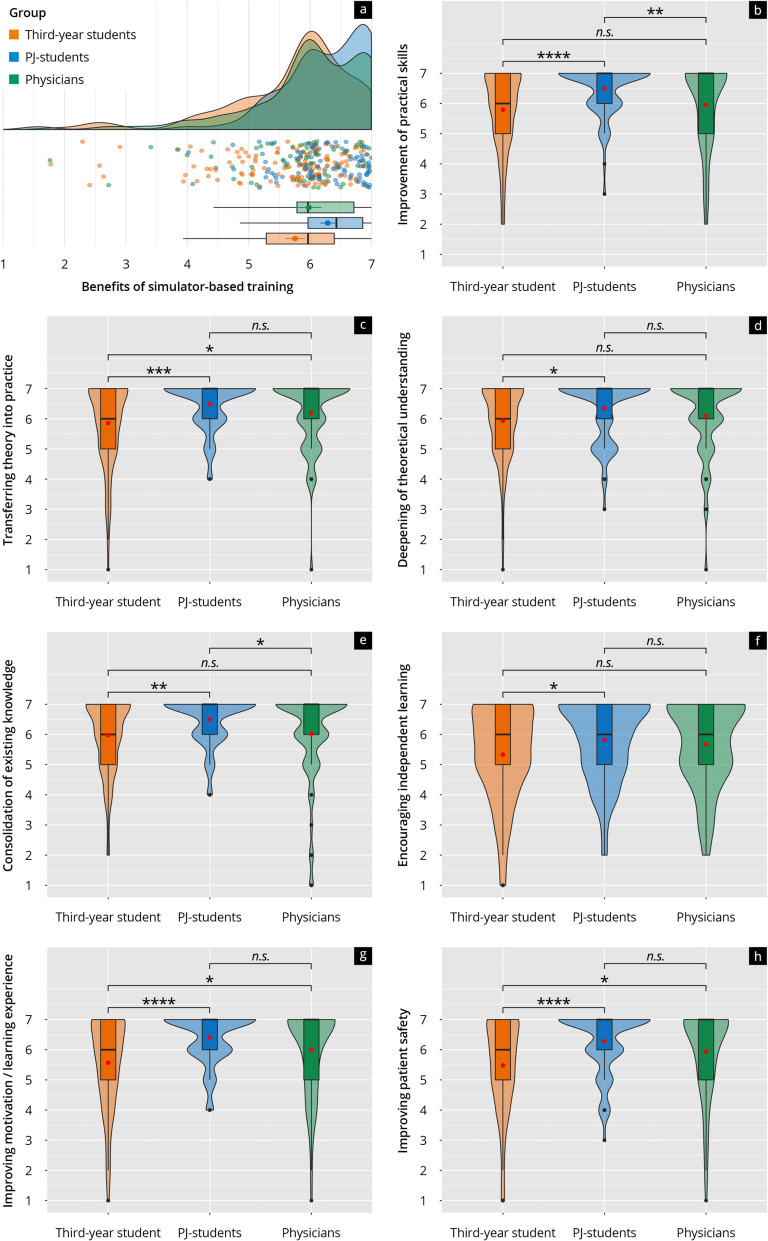
**a** Rain-plot of the mean subitem ratings for the main feature “Benefits of simulator-based training” on a Likert scale (1–7). The distributions for third-year students (orange), PJ students (blue), and physicians (green) are represented by density curves, with individual rating points displayed as a scatter below these curves. Vertical lines within each color group indicate the median and quartiles. **b**–**h** Violin plots showing the distribution of subitem ratings on a Likert scale for third-year students (orange), PJ students (blue), and physicians (green). The *p*-values as results of post-hoc tests after comparing the individual groups are indicated above the brackets, with the corresponding asterisks (* *p* < 0.05, ** *p* < 0.01, *** *p* < 0.001, **** *p* < 0.0001, n.s. = not significant)

When evaluating the training effectiveness, all groups reported an improvement in practical skills and the transfer of theoretical knowledge into practice, with median scores between 4.79 (third-year students) and 6.49 (PJ students) for practical skills (Fig. 3b) and between 5.86 (third-year students) and 6.19 (physicians) for transferring theory into practice (Fig. 3c). The deepening of theoretical understanding (Fig. 3d), the consolidation of existing knowledge (Fig. 3e), the power to encourage independent learning (Fig. 3f) and to improve motivation and the learning experience (Fig. 3g) as well as the contribution to improving patient safety (Fig. 3h) also received consistently high ratings with PJ students and doctors scoring significantly higher in some areas (see Fig. 3).

**Fig. 4:**
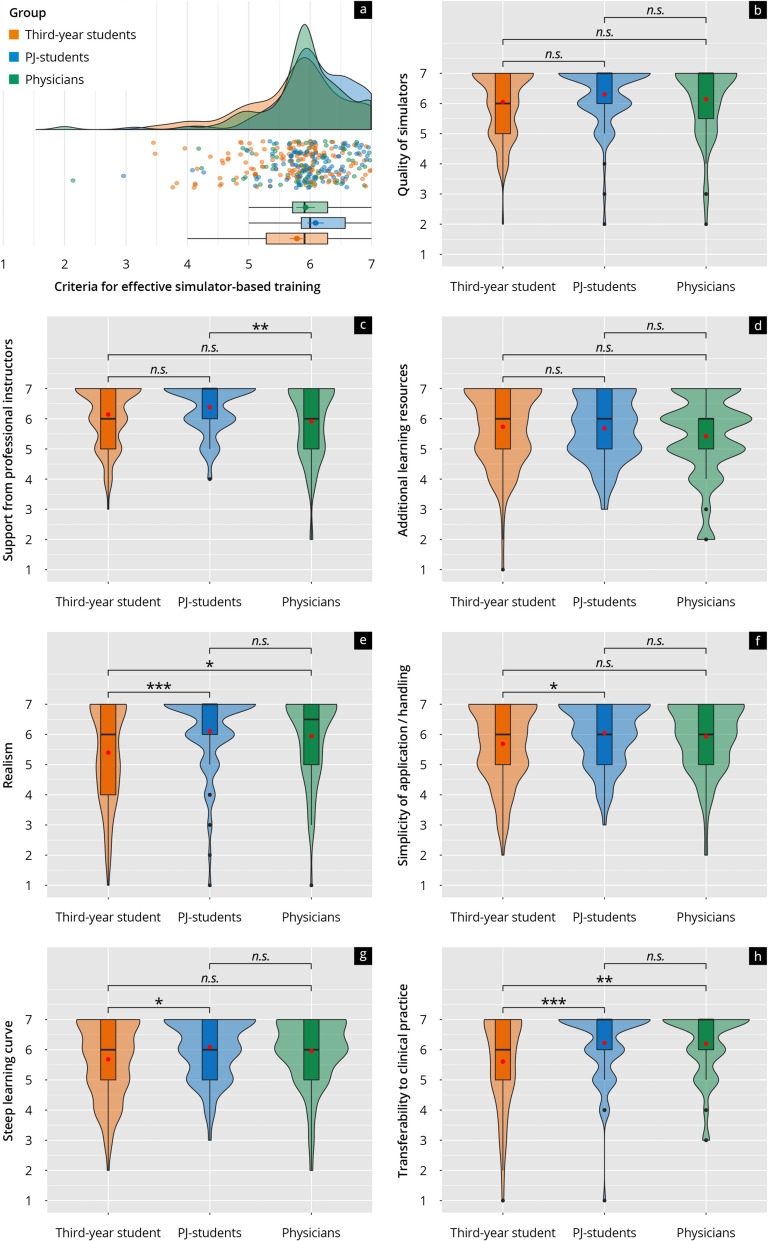
**a** Rain-plot of the mean subitem ratings for the main feature “Criteria for a good quality of simulator-based training” on a Likert scale (1–7). The distributions for third-year students (orange), PJ students (blue), and physicians (green) are represented by density curves, with individual rating points displayed as a scatter below these curves. Vertical lines within each color group indicate the median and quartiles. **b**–**h** Violin plots showing the distribution of subitem ratings on a Likert scale for third-year students (orange), PJ students (blue), and physicians (green). The *p*-values as results of post-hoc tests after comparing the individual groups are indicated above the brackets, with the corresponding asterisks (* *p* < 0.05, ** *p* < 0.01, *** *p* < 0.001, n.s. = not significant)

As depicted in Fig. 4b, the quality of simulators was consistently regarded as crucial for effective training, receiving scores of 5 points or higher across all groups. Support from professional instructors received average scores ranging from 4.92 to 5.38 (Fig. 4c). At the same time, additional learning resources were rated slightly higher, with mean scores between 5.42 and 5.73, showing no significant differences between the groups (Fig. 4d). Realism achieved scores between 5.4 and 6.11 (Fig. 4e), with significantly higher ratings from PJ students and physicians than third-year students. The simplicity of handling or application (Fig. 4f) and the learning curve were rated similarly across all groups, with values between 4.68 and 5.08. Still, PJ students gave significantly higher ratings than third-year students (Fig. 4g). The criterion of transferability to clinical practice was widely endorsed by all groups, with physicians and PJ students rating it significantly higher than third-year students (Fig. 4h).

#### Challenges, limitations, and reasons against the use of simulator-based training

**Fig. 5:**
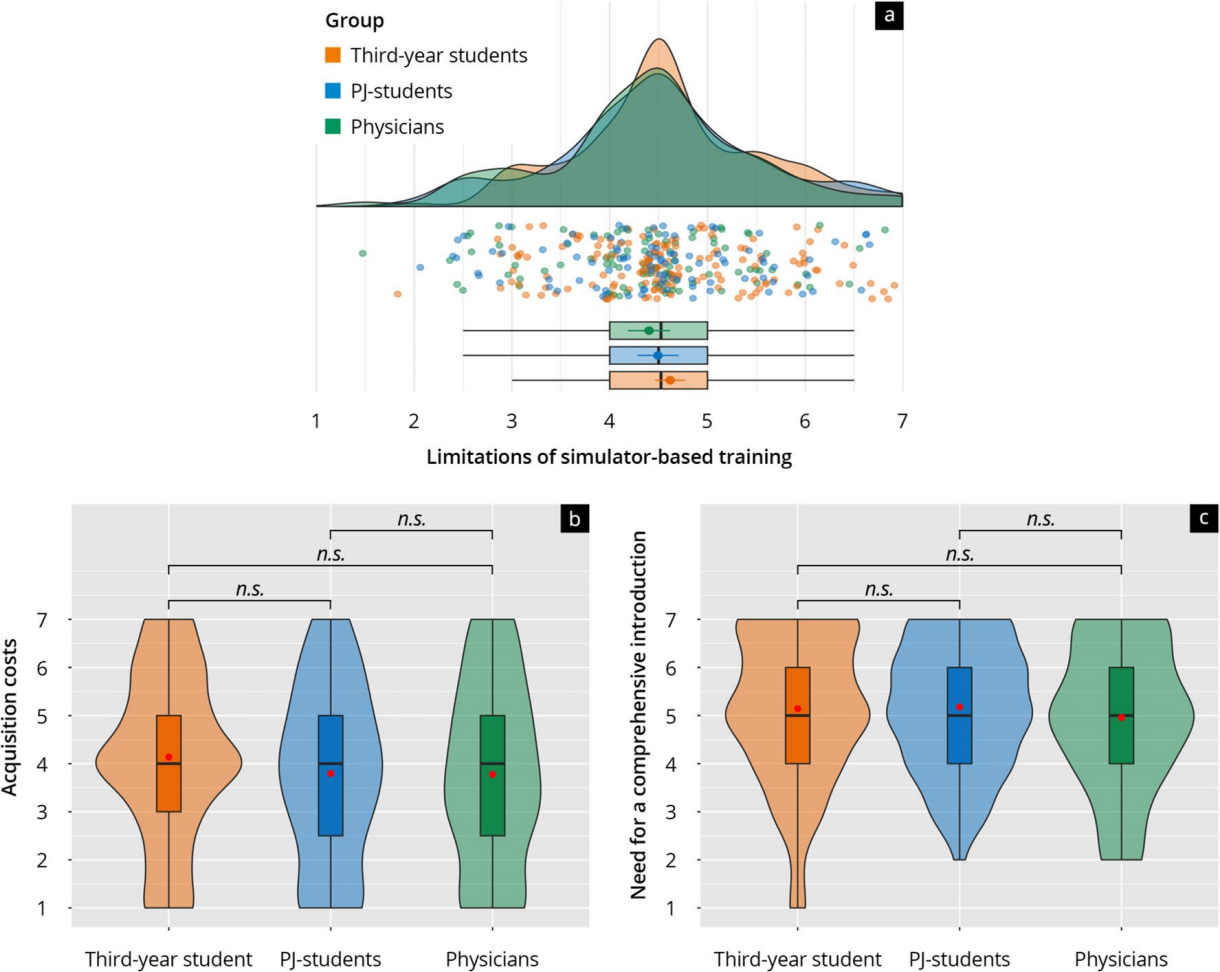
**a** Rain-plot of the mean subitem ratings in the category “Limitations of simulator-based training” on a Likert scale (1–7). **b** The violin plot shows the distribution of ratings for the subitem “high acquisition costs” for third-year students (orange), PJ students (blue), and physicians (green). **c** The violin plot shows the distribution of ratings for the subitem “lack of instruction in the use of the simulator” for third-year students (orange), PJ students (blue), and physicians (green). The *p*-values as results of post-hoc tests after comparing the individual groups are indicated above the brackets (n.s. = not significant)

Regarding the challenges of simulator-based training, 38% of third-year students, 47% of PJ students, and 49% of physicians cited limited budgets as a central issue, with no significant differences between the groups (*p* = 0.1857). The high acquisition costs were perceived as a significant weakness, especially by third-year students (average score of 4.13). PJ students and physicians viewed this aspect less critically, with scores of 3.8 and 3.77, respectively (Fig. 5b). The lack of facilities or teaching staff was similarly rated across groups (*p* = 0.06655; *p* = 0.08881 respectively). The need for a comprehensive introduction to the use of simulators was seen as a potential area for improvement across all groups (Fig. 5c). However, significant differences emerged when considering the lack of teaching concepts or responsibilities as hindrances (*p* < 0.01477 or *p* < 0.01909), with physicians often citing these points as reasons against the use of simulators (see Supplementary Materials Table S5). Despite these barriers, respondents emphasized that none of these factors argue against using simulators.

#### Contact with ultrasound simulators and demand for the use of ultrasound

##### Simulators in specific fields

While about 62% of third-year students and 61% of PJ students and physicians reported having had prior contact with ultrasound simulators (*p* = 0.239), the survey found that experience with the particular ultrasound simulator system used in the course was in the single-digit percentage range for all groups (*p* = 0.3227). When asked about the demand for ultrasound simulators in specific fields, significant differences were noted when assessing their use in gynecology. Here, most students in both groups expressed the need for increased use, while only 27% of physicians supported this notion, and 52% rejected it (*p* < 0.001). A similar picture emerged regarding the demand for their use in vascular punctures (*p* = 0.001072). The demand for their use in intestinal sonography was lower in all groups (*p* = 0.8489). Conversely, the majority of respondents in all groups expressed a demand for their use in abdominal sonography (*p* = 0.005698), emergency sonography (*p* = 0.001245), and echocardiography (*p* = 0.1896) (see Supplementary Materials Table S6).

#### Evaluation of ultrasound simulator training and evaluation of the used

##### Ultrasound simulator

**Fig. 6:**
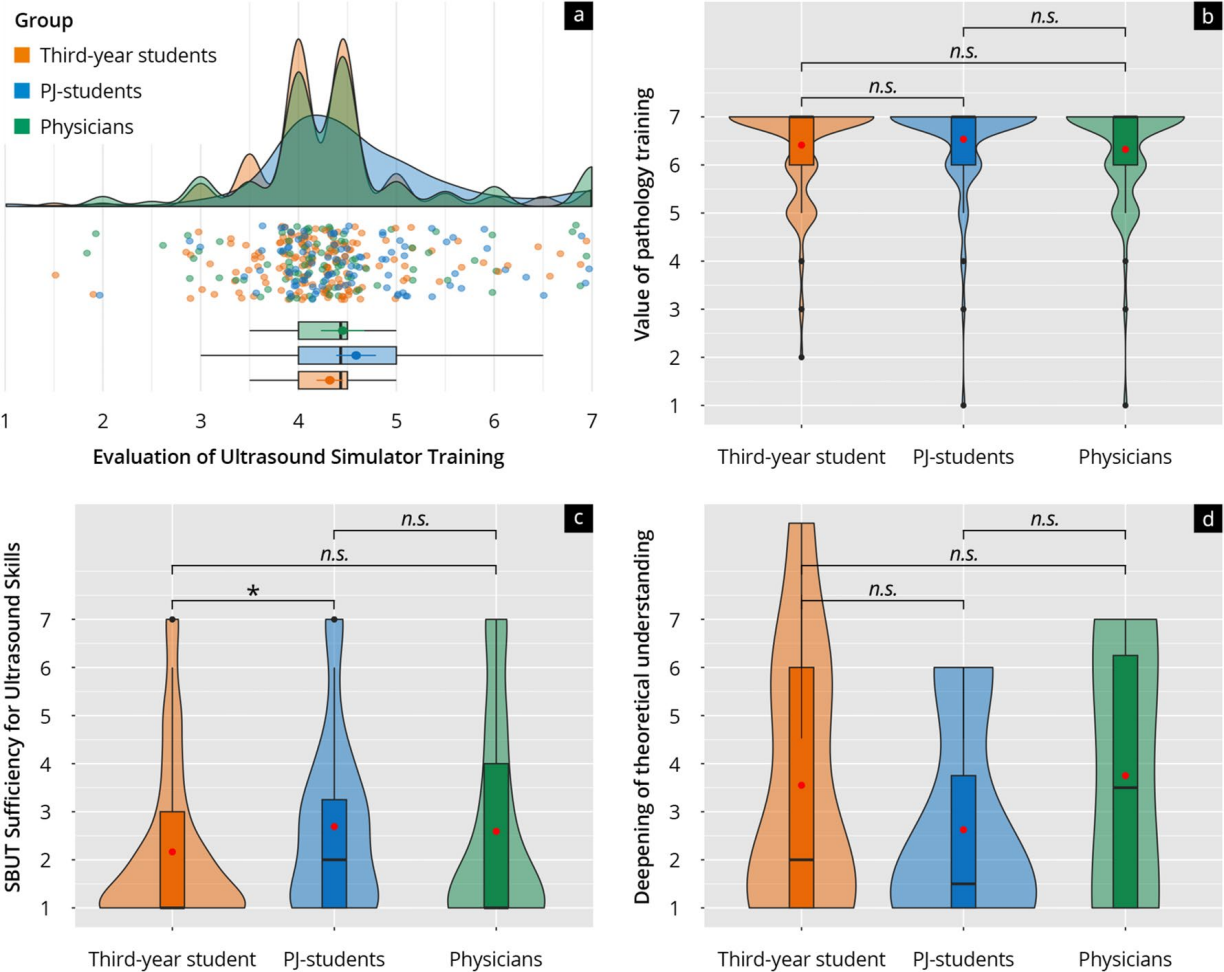
**a** Rain-plot of the mean subitem ratings for the main feature “Evaluation of Ultrasound Simulator Training” on a Likert scale (1–7). The distributions for third-year students (orange), PJ students (blue), and physicians (green) are represented by density curves, with individual rating points displayed as a scatter below these curves. Vertical lines within each color group indicate the median and quartiles. **b** The violin plot shows the distribution of ratings for the subitem “value of pathology training” on a Likert scale for third-year students (orange), PJ students (blue), and physicians (green). **c** Violin plot showing the distribution of ratings for the subitem “Simulator-based Ultrasound-Training (SBUT) Sufficiency for Ultrasound Skills” on a Likert scale for third-year students (orange), PJ students (blue), and physicians (green). **d** The violin plot shows the distribution of ratings for the subitem “Deepening of theoretical understanding” on a Likert scale for third-year students (orange), PJ students (blue), and physicians (green). After comparing the individual groups, the *p*-values as post-hoc test results are indicated above the brackets, with the corresponding asterisks (* *p* < 0.05, n.s. = not significant)

The evaluation of ultrasound simulator training and the used simulator showed that all respondent groups highly valued pathology training for clinical practice (Fig. 6b), with average values from 6.31 (physicians) to 6.54 (PJ students). However, the view that training on the ultrasound simulator alone is sufficient to learn examination techniques (Fig. 6c) did not find significant support in any group (average values 2.17 to 2.69). Deepening of theoretical understanding received relatively low agreement ratings across all groups (Fig. 6d).

**Fig. 7:**
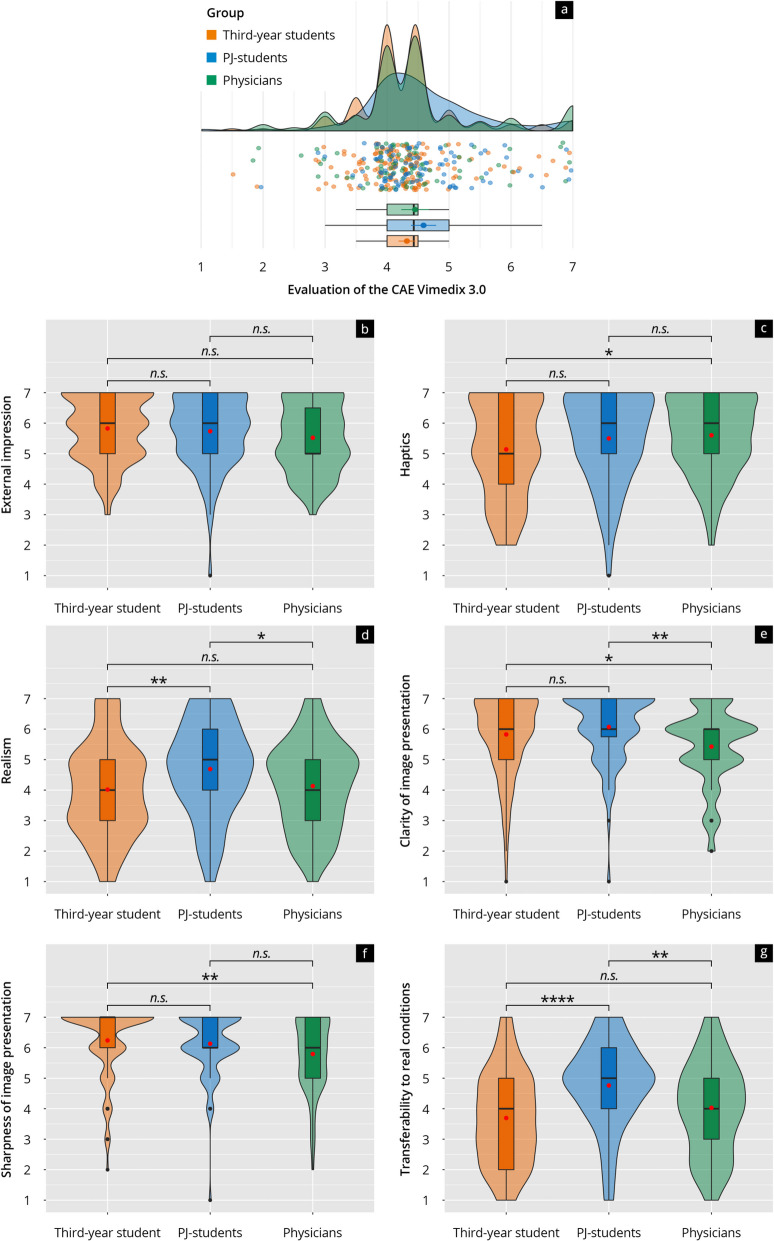
**a** Rain-plot of the mean subitem ratings for the main feature “Evaluation of the CAE Vimedix 3.0 simulator “ on a Likert scale (1–7). The distributions for third-year students (orange), PJ students (blue), and physicians (green) are represented by density curves, with individual rating points displayed as a scatter below these curves. Vertical lines within each color group indicate the median and quartiles. **b**–**g** Violin plots showing the distribution of subitem ratings on a Likert scale for third-year students (orange), PJ students (blue), and physicians (green). The *p*-values as results of post-hoc tests after comparing the individual groups are indicated above the brackets, with the corresponding asterisks (* *p* < 0.05, ** *p* < 0.01, **** *p* < 0.0001, n.s. = not significant)

In the specific evaluation of the ultrasound simulator, physical characteristics (Fig. 7b), the feel of the ultrasound probe (Fig. 7c), and the sharpness (Fig. 7f) and clarity (Fig. 7e) of image presentation on the laptop were rated positively across all groups. On the other hand, realism (Fig. 7d) and the direct transferability of the simulator's display to real conditions (Fig. 7g) received lower ratings (4.02 to 4.69) (see Fig. 7).

**Fig. 8:**
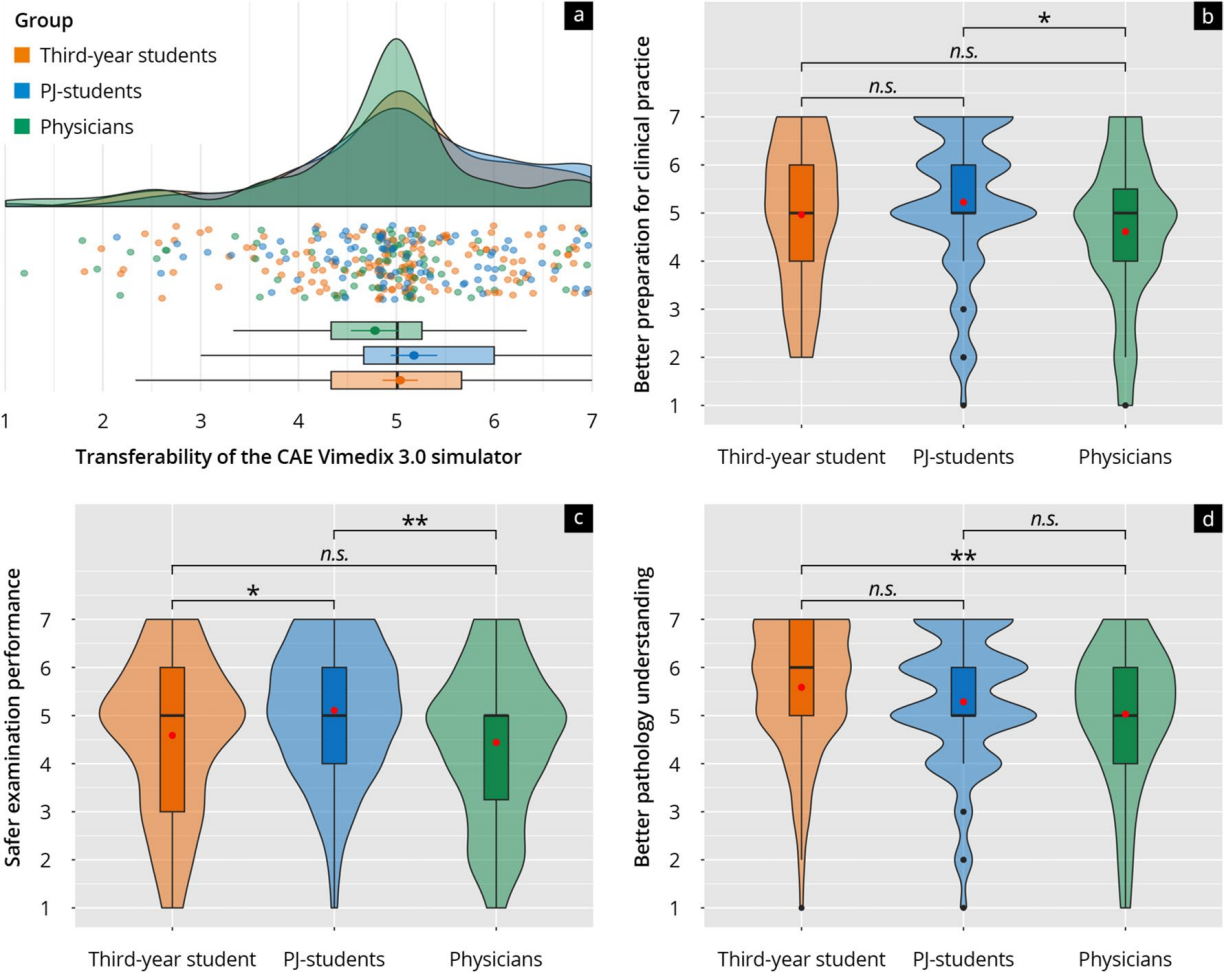
**a** Rain-plot of the mean subitem ratings for the main feature “Transferability of the CAE Vimedix 3.0 simulator “ on a Likert scale (1–7). The distributions for third-year students (orange), PJ students (blue), and physicians (green) are represented by density curves, with individual rating points displayed as a scatter below these curves. Vertical lines within each color group indicate the median and quartiles. **b**–**d** Violin plots showing the distribution of subitem ratings on a Likert scale for third-year students (orange), PJ students (blue), and physicians (green). The *p*-values as results of post-hoc tests after comparing the individual groups are indicated above the brackets, with the corresponding asterisks (* *p* < 0.05, ** *p* < 0.01, n.s. = not significant)

The transferability of the simulator training to general clinical practice in terms of better preparation or everyday clinical practice was rated highest by PJ students with an average value of 5.23, followed by third-year students (4.97) and physicians (4.61) (Fig. 8b). Similarly, the evaluation of safer examination performance through simulator training was also highlighted, with physicians giving lower approval than the other two groups, with an average score of 4.44 (Fig. 8c). Also, the aspect of better pathology understanding through simulator-based ultrasound training was given high average scores across all groups, with the approval in the physicians’ group being lower than in the other two groups (5.03) (Fig. 8d).

## Discussion

This study aimed to comprehensively understand the perspectives and experiences of students and physicians regarding the acceptance and implementation of simulator-based training in medical education. Specifically, it explored the perceived benefits and challenges of simulator training, focusing on ultrasound simulators, and evaluated its integration into medical education curricula.

Our findings highlight that while simulator-based training is widely recognized and utilized in medical education, significant variations exist between different medical fields, levels of training and user groups. These differences underline the need for targeted strategies to enhance the integration of simulator training into curricula. Specifically, the results emphasize challenges in implementing ultrasound simulator training effectively, the need for standardized teaching methods, and the importance of qualified teaching staff to optimize training outcomes. Addressing these aspects is essential to ensure a comprehensive and high-quality education for future generations of physicians, ultimately contributing to improved patient care.

### General attitude towards simulator-based training

Our survey revealed significant differences in the exposure to and acceptance of simulator-based training among third-year students, PJ students, and physicians. As shown in Sect. "Experiences with Simulator-Based Training", PJ students reported the highest exposure level, likely attributable to their participation in advanced clinical skills courses. In contrast, third-year students and physicians demonstrated lower levels of engagement with simulator-based training. The most common applications of simulators were in fields such as airway management, emergency medicine, and ultrasound, whereas their use in pediatrics and gynecology was notably less frequent. These findings are consistent with prior studies that emphasize the successful integration of simulators into these high-priority areas [34–37].

The observed differences in perception and usage likely reflect the distinct needs and training priorities of the surveyed groups. Students in earlier stages of medical education, such as third-year and PJ students, focus on acquiring foundational skills in a structured and supportive environment. In contrast, practicing physicians may prioritize targeted and specialized training that aligns more closely with their clinical responsibilities and day-to-day practice. Despite recognized benefits, these divergent priorities likely contribute to the lower perceived relevance of simulator-based training among practicing physicians. Additionally, the differences in exposure underscore potential gaps in integrating simulator-based training across curricula. While students, particularly PJ students, often view simulator-based training as a valuable tool for skill acquisition, physicians may view it as less critical, given their reliance on hands-on patient interactions and prior experience.

### Challenges in supervision and integration of simulator-based training into mandatory education

The survey results highlighted significant disparities in integrating simulator-based training into mandatory curricula, particularly among physicians. Only 14% of physicians reported that simulator-based training was incorporated into their required training programs, a figure significantly lower than the rates among students, especially PJ students. This discrepancy underscores critical gaps in the adoption of simulation technologies across different stages of medical education.

Differences influence the uneven integration in the focus of medical curricula. For students, particularly PJ students, simulator-based training often serves as a foundational component in advanced clinical skills courses, offering structured opportunities for hands-on practice. In contrast, physicians frequently rely on practical experience and traditional methods for skill acquisition, resulting in limited curricular integration of simulator-based training for continuing education. Despite its potential benefits, these gaps highlight the undervaluation of simulation-based learning in clinical practice.

As noted in Sect. "Instructors, Assessment of Learning Success at Simulators, and Expansion of Simulation Centers", peer-assisted learning (PAL) emerged as a standard method of instruction among students, especially PJ students. PAL has been recognized for its collaborative benefits and positive reception among trainees, but it also has limitations. Nunnink et al. emphasize that PAL benefits peer teachers more than learners and that professional supervision is critical to maintaining technical accuracy and educational quality [36]. Our findings align with this perspective, underscoring the need for qualified instructors to provide oversight and ensure the effectiveness of training sessions. Such supervision is necessary for the quality of simulator training experiences to remain high, particularly in settings where resources are limited.

In addition to the structural challenges, respondents also emphasized the need for specific content delivery tailored to the requirements of different learner groups. As highlighted in Sect. "Integration into Mandatory Training and Specific Content Delivery through", students sought foundational training in emergency medicine and airway management, whereas physicians favored specialized scenarios aligned with their clinical expertise. This expectation divergence underscores the importance of curricular reforms that integrate simulator training into mandatory programs and address learners' diverse content needs.

Proposals for the integration of simulator-based training, as outlined in the AMEE Guide No. 82, emphasize the importance of aligning simulation curricula with the specific needs of learners. Motola et al. highlight that effective integration of simulation into medical education requires careful planning, needs-oriented learning objectives, and the support of professional feedback and reflection, as well as resource utilization to optimize learning success [35]. Similarly, McGaghie et al. stress that mastery learning relies on systematically assessing participants' performance to evaluate outcomes and adapt training accordingly [38]. With clear guidelines, institutions can develop cohesive training programs aligning with educational objectives and clinical requirements.

Addressing these challenges requires a multifaceted approach:

Expanding simulator-based training in mandatory curricula for physicians.

Developing specific content frameworks tailored to different learner groups.

Ensuring the consistent supervision of simulator training by qualified instructors.

Establishing institutional guidelines to support standardized implementation and evaluation.

By focusing on these areas, medical education can better leverage the potential of simulation technologies to enhance learning outcomes and bridge training gaps across all education stages.

### Benefits and the necessity of adaptive teaching methods

The findings from Sects. "Perceived Benefits and Challenges of Simulator-Based Training" and "Challenges, Limitations, and Reasons Against the Use of Simulator-Based Training" highlight the diverse requirements and challenges associated with simulator-based training. While the perceived benefits of simulators—such as improved clinical skills, enhanced theory-to-practice transfer, and increased learner autonomy—were widely acknowledged, significant limitations remain. These include high costs, technical constraints, and limited realism, all impacting the perceived applicability of simulators to clinical practice.

Addressing these challenges requires a shift towards adaptive teaching methods tailored to the distinct needs of different learner groups. As highlighted in Sect. "Perceived Benefits and Challenges of Simulator-Based Training", students preferred standardized foundational training, particularly in emergency medicine and airway management. In contrast, physicians emphasized the importance of specialized scenarios that align with their clinical expertise. This divergence underscores the necessity of developing curricula that cater to both beginner and advanced learners, ensuring that training content is relevant and practical.

Khamis et al. propose a structured six-step approach to establishing simulator-based training. This approach involves conducting a needs analysis, setting measurable learning objectives, selecting appropriate teaching strategies, and implementing and evaluating the curriculum [39]. These principles align with our findings, emphasizing the importance of designing training programs that are both needs-oriented and adaptable.

Another critical aspect is improving the realism of simulation training. As noted in Sect. "Challenges, Limitations, and Reasons Against the Use of Simulator-Based Training", respondents frequently cited the lack of realism as a barrier to effective learning. Enhancing the fidelity of simulation technologies and aligning training scenarios with real-world clinical demands are essential to bridge this gap. This includes integrating high-fidelity simulators and developing scenarios that closely replicate clinical environments.

Despite the challenges, simulator-based training offers significant long-term benefits. Fletcher et al. highlight that while simulators may have high initial costs, their potential to improve safety, efficiency, and overall learning outcomes justifies the investment [40]. Similarly, our findings suggest that many respondents viewed budget constraints as secondary concerns compared to the broader benefits of simulator training. This highlights the need for strategic resource allocation to ensure cost-effective implementation.

Finally, the success of simulator-based training relies not only on technology but also on the availability of well-trained instructors and comprehensive teaching materials. As Motola et al. emphasize, effective integration of simulation into medical education requires professional feedback, resource utilization, and continuous program assessment [35]. By adopting these strategies, medical education can leverage the full potential of simulation technologies to enhance learning outcomes and address the diverse needs of its learners.

### Ultrasound simulator training

In our study, most respondents across all groups reported having prior experience with ultrasound simulators, as highlighted in Sect. "Contact with Ultrasound Simulators and Demand for the Use of Ultrasound". However, engagement with the specific ultrasound system used in the study was relatively low, with usage rates falling into the single-digit percentages for all groups. This discrepancy may be attributed to the wide range of available ultrasound simulators, as seen in previous studies [22]. This diversity reinforces the necessity for standardization and comparability to ensure consistent training results.

Standardization should encompass the manufacturers' equipment and the didactic design of courses to maintain consistent quality across various training programs. Simulation-based training has also proven to be an effective method for acquiring ultrasound skills, significantly enhancing the clinical capabilities of resident physicians [41–43]. Comprehensive needs analyses have emphasized the importance of developing standardized simulation-based training programs for different ultrasound-based techniques [44]. Various simulation-based training modules, including basic ultrasound techniques, ultrasound-guided interventions, interpretation skills, professionalism, and team training, have already been integrated into pre- and postgraduate medical education curricula [45, 46].

Our findings also indicate that practical ultrasound simulator exercises are crucial for skill acquisition, particularly before applying these procedures to patients. As highlighted in Sect. "Evaluation of Ultrasound Simulator Training and Evaluation of the Used", respondents evaluated the ultrasound simulator's realism and clinical applicability, identifying strengths and areas for improvement. This aligns with earlier research emphasizing the importance of practicing on simulators before patient contact [47]. The development of comprehensive training programs and practical exercises is essential. This also requires tools to measure learning success. The Direct Observation of Procedural Skills (DOPS) approach has proven effective in this context. It can serve as a valuable way of monitoring the level of success in applying practical skills in connection with ultrasound simulators [48].

To optimize the use of ultrasound simulators in medical training, it is essential to regularly assess and adapt both the training content and the simulators themselves to meet the evolving needs of different learner groups. This includes enhancing the realism and usability of simulators and ensuring that the training scenarios are relevant to the clinical context in which learners will eventually operate. By doing so, we can bridge the gap between simulation and real-life clinical practice, thereby improving overall training quality and patient care outcomes.

### Requirements and expectations for ultrasound simulators

The findings from Sects. "Contact with Ultrasound Simulators and Demand for the Use of Ultrasound" and "Evaluation of Ultrasound Simulator Training and Evaluation of the Used" highlight the diverse requirements and expectations for ultrasound simulators across different learner groups. While the simulators in our courses received positive feedback, evaluations of specific features such as realism and usability revealed significant variability. This underscores the need to tailor simulators to the needs of their target audience, ensuring that students and physicians benefit from practical and relevant training. Aspects like realism and direct applicability to real-life conditions received lower scores across the board, aligning with perspectives that suggest simulation equipment selection should prioritize the training program’s specific objectives, the learner demographic, and the educational context over mere realism [49].

One critical aspect is aligning simulation equipment with training program objectives. Previous studies, including Ostergaard et al., have highlighted that no single simulator can universally meet all needs. Their evaluation of virtual reality simulators for abdominal sonography demonstrated that while all systems offered substantial learning benefits, preferences varied depending on user requirements, such as image fidelity, user-friendliness, and overall satisfaction [22]. This variability highlights the importance of flexible, target-specific training designs that address the unique demands of each learner group.

Students, particularly those in earlier medical education, often prioritize foundational skills, such as image acquisition and interpretation, while physicians focus on advanced diagnostic challenges and specialized applications. These differences emphasize the need for training programs to adapt their content and methodologies to suit their participants' experience levels and learning goals.

Moreover, regular assessment and revision of training curricula are essential to keeping pace with medical learners’ evolving needs and expectations. As highlighted in Sect. "Evaluation of Ultrasound Simulator Training and Evaluation of the Used", respondents identified a strong demand for improved realism and applicability of simulators to real-life clinical scenarios. Addressing these expectations requires collaboration between educators and manufacturers to refine simulator technical specifications and teaching strategies.

Medical education can ensure that ultrasound simulators effectively enhance clinical competencies and meet the varied demands of its learners by prioritizing the development of adaptive training materials and incorporating regular feedback from diverse learner groups. This approach maximizes the impact of simulation technologies and aligns training programs with the overarching goal of improving patient care through high-quality education.

### Limitations

The study’s potential self-selection bias, arising from its conduct within specific ultrasound courses tailored to different target groups, warrants discussion. The variation in course content and teaching methods across abdominal sonography for physicians, ultrasound training for third-year students, and clinical skills for PJ students may have impacted participants’ perceptions and evaluations of simulator-based training. Additionally, the courses’ specific focus could have influenced participants’ prior knowledge and expectations about simulator training. Another limitation is the heterogeneity within the physician group in terms of their medical specialties. While internal medicine and surgery were well represented, other specialties had fewer participants, which may have influenced the generalizability of the findings. Future studies should strive for a more balanced representation of specialties to better reflect the diverse needs and perceptions of simulator-based training across the medical field. The varied experience levels with simulator training, particularly among older physicians, might introduce selective exposure or confirmation biases among all study participants. Furthermore, since physicians’ prior engagements with simulator-based training often occurred earlier in their careers or during pre-training, the potential for a recall bias influencing responses must also be considered.

## Conclusions

Our study reveals notable differences in how medical students and physicians experience and perceive simulator-based training. Despite varying contact points with these instructional approaches across different stages of medical education, all groups acknowledged the benefits of simulation and emphasized its importance for practical application and patient safety. This finding underscores the need to integrate simulator-based training into medical curricula irrespective of the level of education or specialty. A particular focus should be placed on adjusting teaching methods to the needs of different learning groups to ensure comprehensive and practical instruction. Our results further suggest that ultrasound simulator training—particularly within certified courses—represents a valuable adjunct, effectively delivering extensive, sustainable, and standardized ultrasound knowledge and skills.

Further research in this area is necessary to customize teaching concepts specifically to the needs of different target groups. This would optimize teaching methods and promote safe and efficient patient care. This approach is crucial to meeting the challenges of modern medical education while continuously improving the quality of medical care.

## Supplementary Information

Supplementary Material 1.

## Data Availability

Data cannot be shared publicly because of institutional and national data policy restrictions imposed by the Ethics committee since the data contain potentially identifying study participants’ information. Data are available upon request from the Johannes Gutenberg University Mainz Medical Center (contact via weimer@uni-mainz.de) for researchers who meet the criteria for access to confidential data (please provide the manuscript title with your enquiry).
