# Supplementary material for: Reflecting realities: gauging the pulse of simulator-based training in medical minds—resonance of simulator-based ultrasound training in medical education
Source: BMC Med Educ. 2025 May 6;25:664. doi: 10.1186/s12909-025-07198-4 (PMC12057109; doi:10.1186/s12909-025-07198-4)
Supplement: Supplementary file 1 — Supplementary Material 1. [file 12909_2025_7198_MOESM1_ESM.docx]

**Supplementary Materials:**

**Table S1:** *Design and structure of the questionnaire with presentation of the topics and subitems as well as the corresponding scale level.*

| **Topic** | **Subitems** | **Scale** |
| --- | --- | --- |
| Baseline | Age  Sex  rescue personnel 3rd year Student  Practical-year Student  Physician (+Resident) | binary |
| Points of contact with simulator-based training | Points of contact as a participant  Points of contact as an instructor  Points of contact during prior education  Points of contact in other areas | binary |
| Areas of use for simulators | Airway Management  Ultrasound Diagnostics  Gynecology  Emergency Medicine  Pediatrics  In other areas | binary |
| Instructor at Simulators | Students  Physicians  External Partners  Emergency Medical Services Personnel  None | binary |
| Learning Outcome Assessment at the Simulator | Written Test  Practical Exam  No Test  Examinations in Other Formats | binary |
| Expansion of Simulation Centers | Expansion of the Simulation Centers in the last 5 years  Demand for expansion/new establishment | binary |
| Reasons for the use of simulators | Educational purposes  Improvement of training  Trend in didactics/innovation  Cooperation with external partners | binary |
| Reasons against the use of simulators | Limited budget resources  Lack of space  Lack of teaching staff  Absence of teaching concepts  Lack of responsibility | binary |
| Use of simulators in training | Integration of SBT (Simulation-Based Training) into mandatory training  No use in training  Use in preclinical education  Use in clinical education  Use in specialist medical training  Use in continuing education courses  Use in training professions | binary |
| Contents conveyed by simulators | Gynecology  Anesthesiology  Internal Medicine  Surgery | binary |
| Contact with ultrasound simulators | General contact with ultrasound simulators  CAE Vimedix | binary |
| Demand for ultrasound simulators in specific disciplines | Gynecology  Abdominal sonography  Emergency sonography  Vascular access (CVC)  Intestinal sonography  Echocardiography  Other areas | binary |
| Request for the use of simulators | Medical studies  Specialist medical training  Demand for more simulation-based training  Demand for expansion of simulation centers/new establishment | ordinal |
| Benefits of simulator-based training | Improvement of practical skills  Transferring theory into practice  Deepening of theoretical understanding  Consolidation of existing knowledge  Encouraging independent learning  Increasing motivation and improving the learning experience  Safety | ordinal |
| Limitations of simulator-based training | high acquisition costs  need for a comprehensive introduction | ordinal |
| Criteria for effective simulator-based training | Quality of simulators  Support from professional instructors  Additional learning resources  Realism  Simplicity of application / handling  steep learning curve  Transferability to clinical practice | ordinal |
| Evaluation of Simulators | Pathology training  Simulator training only to learn ultrasound examination | ordinal |
| Evaluation of the CAE Vimedix 3.0 | External assessment  Haptics  Reality  Overview  Image clarity  Transfer | ordinal |
| Transferability of the CAE Vimedix 3.0 simulator | Transferability to the real patient situation  Safety in handling  Understanding pathology | ordinal |

**Table S2:** *Exposure to Simulator-based Training and Areas of Use for Simulator-Based Training*

| Group | **clinical medical students** | **internship students** | **Physicians** | **P-Value** |
| --- | --- | --- | --- | --- |
|  | **n= 154 (%)** | **n= 97 (%)** | **n= 92 (%)** |  |
| *Exposure to Simulator-based Training* | | | | |
| Points of contact as a participant  Yes  No  not specified | 90 (58)  56 (36)  8 (6) | 75 (77)  22 (23)  0 (0) | 61 (66)  31 (34)  0 (0) | **0.03685** |
| Point of contact as an instructor  Yes  No  not specified | 7 (5)  137 (89)  8 (6) | 6 (6)  91 (94)  0 (0) | 8 (9)  81 (88)  3 (3) | 0.4538 |
| Points of contact  in preliminary training  Yes  No  not specified | 122 (79)  32 (21)  0 (0) | 69 (71)  28 (29)  0 (0) | 88 (96)  4 (4)  0 | **<0.001** |
| Points of contact in other areas  Yes  No  not specified | 147 (95)  7 (5)  0 (0) | 94 (97)  3 (3)  0 (0) | 83 (90)  9 (10)  0 (0) | 0.102 |
|  | **N= 154 (%)** | **N= 97 (%)** | **N= 92 (%)** |  |
| *Areas of Use for Simulator-Based Training* | | | | |
| Use in  Airwaymanagement  Yes  No  not specified | 27 (18)  109 (71)  18 (11) | 54 (56)  35 (36)  8 (8) | 15 (16)  66 (72)  11 (12) | **<0.001** |
| Use in Ultrasound  Yes  No  not specified | 32 (21)  104 (68)  18 (11) | 34 (35)  55 (57)  8 (8) | 7 (8)  74 (80)  11 (12) | **<0.001** |
| Use in gynecology  Yes  No  not specified | 2 (1)  134 (87)  18 (12) | 18 (19)  71 (73)  8 (8) | 2 (2)  79 (86)  11 (12) | **<0.001** |
| Use in emergency medicine  Yes  No  not specified | 18 (12)  118 (77)  18 (11) | 44 (45)  45 (46)  8 (9) | 13 (14)  68 (74)  11 (12) | **<0.001** |
| Use in pediatrics  Yes  No  not specified | 4 (3)  132 (86)  18 (11) | 8 (8)  81 (84)  8 (8) | 4 (4)  77 (84)  11 (12) | 0.1361 |
| Use in other areas  Yes  No  not specified | 12 (8)  124 (81)  18 (11) | 6 (6)  83 (86)  8 (8) | 5 (5)  76 (83)  11 (12) | 0.7329 |

**Table S2:** *Instructors, Assessment of Learning Success at Simulators, and Expansion of Simulation Centers*

|  | **clinical medical students** | **internship students** | **Physicians** | **P-Value** |
| --- | --- | --- | --- | --- |
|  | **n = 154(%)** | **N= 97 (%)** | **N= 92 (%)** |  |
| *Instructors in Simulation Centers* | | | | |
| Students as instructors  Yes  No  not specified | 65 (42)  71 (46)  18 (12) | 62 (64)  27 (28)  8 (8) | 12 (13)  69 (75)  11 (12) | **<0.001** |
| Physicians as instructors  Yes  No  not specified | 41 (27)  95 (62)  18 (11) | 60 (62)  29 (30)  8 (8) | 25 (27)  56 (61)  11 (12) | **<0.001** |
| External partners as  instructors  Yes  No  not specified | 13 (8)  123 (80)  18 (12) | 15 (15)  74 (76)  8 (9) | 2 (2)  79 (86)  11 (12) | **0.006944** |
| Emergency medical personnel as instructors  Yes  No  not specified | 24 (16)  112 (73)  18 (11) | 24 (25)  65 (67)  8 (8) | 8 (9)  73 (79)  11 (12) | **0.01535** |
| No instructors  Yes  No  not specified | 0 (0)  136 (88)  18 (12) | 1 (1)  88 (91)  8 (8) | 3 (3)  78 (85)  11 (12) | 0.06619 |
|  | **clinical medical students** | **internship students** | **Physicians** | **P-Value** |
|  | **N= 154 (%)** | **N= 97 (%)** | **N= 92 (%)** |  |
| *Assessment of Learning Success at Simulators* | | | | |
| Written exam  Yes  No  not specified | 11 (7)  125 (81)  18 (12) | 11 (11)  78 (80)  8 (9) | 2 (2)  79 (86)  11 (12) | 0.05615 |
| Practical exam  Yes  No  not specified | 57 (37)  79 (51)  18 (12) | 26 (27)  63 (65)  8 (8) | 9 (10)  72 (78)  11 (12) | **<0.001** |
| No exam  Yes  No  not specified | 50 (32)  86 (56)  18 (12) | 53 (55)  36 (37)  8 (8) | 31 (34)  50 (54)  11 (12) | **0.0017** |
| Exam in other formats  Yes  No  not specified | 6 (4)  130 (84)  18 (12) | 6 (6)  83 (86)  8 (8) | 3 (3)  78 (85)  11 (12) | 0.617 |
|  | **clinical medical students** | **internship students** | **Physicians** | **P-Value** |
|  | **n = 154 (%)** | **N= 97 (%)** | **N= 92 (%)** |  |
| *Expansion of Simulation Centers* | | | | |
| Expansion of simulation centers in the last 5 years? Yes  No  not specified | 35 (23)  20 (13)  99 (64) | 31 (32)  1 (1)  65 (67) | 6 (7)  14 (15)  72 (78) | **<0.001** |

**Table S4:** *Benefits of Simulator-Based Training*

|  | **clinical medical students** | **internship students** | **Physicians** | **P-Value** |
| --- | --- | --- | --- | --- |
|  | **N= 154 (%)** | **N= 97 (%)** | **N= 92 (%)** |  |
| Educational purposes  Yes  No  not specified | 98 (64)  38 (25)  18 (11) | 81 (84)  8 (8)  8 (8) | 38 (41)  43 (47)  11 (12) | **<0.001** |
| Improvement of education  Yes  No  not specified | 87 (56)  49 (32)  18 (12) | 75 (77)  14 (14)  8 (8) | 38 (41)  43 (47)  11 (12) | **<0.001** |
| Trend in didactics/innovation Yes  No  not specified | 48 (31)  88 (57)  18 (12) | 37 (38)  52 (54)  8 (8) | 9 (10)  72 (78)  11 (12) | **<0.001** |
| Cooperation with external partners  Yes  No  not specified | 22 (14)  114 (74)  18 (12) | 19 (20)  70 (72)  8 (8) | 8 (9)  73 (79)  11 (12) | 0.1253 |

**Table S5:** *Limitations of Simulator-based Training*

|  | **clinical medical students** | **internship students** | **Physicians** | **P-Value** |
| --- | --- | --- | --- | --- |
|  | **n 154 (%)** | **N 97 (%)** | **N 92 (%)** |  |
| limited budget resources  Yes  No  not specified | 59 (38)  77 (50)  18 (12) | 46 (47)  43 (44)  8 (9) | 45 (49)  36 (39)  11 (12) | 0.1857 |
| lack of space  Yes  No  not specified | 19 (12)  117 (76)  18 (12) | 23 (24)  66 (68)  8 (8) | 13 (14)  68 (74)  11 (12) | 0.06655 |
| Shortage of teaching staff  Yes  No  not specified | 41 (27)  95 (62)  18 (11) | 38 (39)  51 (53)  8 (8) | 34 (37)  47 (51)  11 (12) | 0.08881 |
| Absence of teaching concepts  Yes  No  not specified | 32 (21)  104 (68)  18 (12) | 25 (26)  64 (66)  8 (8) | 34 (37)  47 (51)  11 (12) | **0.01477** |
| Lack of responsibilities  Yes  No  not specified | 19 (12)  117 (76)  18 (12) | 17 (18)  72 (74)  8 (8) | 24 (26)  57 (62)  11 (12) | **0.01909** |

**Table S6:** *Integration into Mandatory Training and Specific Content Delivery through Simulator-based Training*

|  | **clinical medical students** | **internship students** | **Physicians** | **P-Value** |
| --- | --- | --- | --- | --- |
|  | **N= 154(%)** | **N= 97 (%)** | **N= 92 (%)** |  |
| *Integration of Simulator-based Training into Mandatory Training* | | | | |
| Integration of simulator-based training into mandatory training Yes  No  not specified | 38 (25)  56 (36)  60 (39) | 47 (48)  29 (30)  21 (22) | 13 (14)  55 (60)  24 (26) | **<0.001** |
| No use in training  Yes  No  not specified | 15 (10)  114 (74)  25 (16) | 5 (5)  80 (82)  12 (13) | 31 (34)  43 (47)  18 (19) | **<0.001** |
| Use in the preclinical phase  Yes  No  not specified | 3 (2)  126 (82)  25 (16) | 8 (8)  77 (79)  12 (13) | 9 (10)  65 (71)  18 (20) | **<0.001** |
| Use in the clinic  Yes  No  not specified | 87 (56)  42 (27)  25 (17) | 73 (75)  12 (12,5)  12 (12,5) | 26 (28)  48 (52)  18 (20) | **<0.001** |
| Use in the  specialist training Yes  No  not specified | 6 (4)  123 (80)  25 (16) | 7 (7)  78 (80)  12 (13) | 17 (18)  57 (62)  18 (20) | **0.000156** |
| Use in training courses  Yes  No  not specified | 30 (19)  99 (64)  25 (16) | 26 (27)  59 (61)  12 (12) | 20 (22)  54 (59)  18 (19) | 0.487 |
| Use in training occupations  Yes  No  not specified | 17 (11)  112 (73)  25 (16) | 17 (18)  68 (70)  12 (12) | 2 (2)  72 (78)  18 (20) | **0.004254** |
|  | **clinical medical students** | **internship students** | **Physicians** | **P-Value** |
|  | **N= 154 (%)** | **n = 97(%)** | **N= 92 (%)** |  |
| *Specific Content Delivery through Simulator-based Training* | | | | |
| Contents of gynecology  Yes  No  not specified | 11 (7)  118 (77)  25 (16) | 36 (37)  49 (51)  12 (12) | 6 (7)  68 (74)  18 (19) | **<0.001** |
| Contents of  anesthesiology  Yes  No  not specified | 34 (22)  95 (62)  25 (16) | 70 (72)  15 (15)  12 (13) | 29 (32)  45 (49)  18 (20) | **<0.001** |
| Contents of internal medicine  Yes  No  not specified | 88 (57)  41 (27)  25 (16) | 52 (54)  33 (34)  12 (12) | 24 (26)  50 (54)  18 (20) | **<0.001** |
| Contents of surgery  Yes  No  not specified | 17 (11)  112 (73)  25 (16) | 48 (49)  37 (38)  12 (13) | 12 (13)  62 (67)  18 (20) | **<0.001** |

**Table S7:** *Contact with Ultrasound Simulators and Demand for the Use of Ultrasound Simulators in Specific Fields*

|  | **clinical medical students** | **internship students** | **Physicians** | **P-Value** |
| --- | --- | --- | --- | --- |
|  | **n = 154 (%)** | **N= 97 (%)** | **N= 92 (%)** |  |
| Contact with ultrasound simulators Yes  No  not specified | 96 (62)  25 (16)  33 (12) | 59 (61)  25 (26)  13 (13) | 56 (61)  14 (15)  22 (24) | 0.239 |
| Contact with CAE-Vimedix  Yes  No  not specified | 10 (6)  115 (75)  29 (19) | 5 (5)  80 (82)  12 (13) | 2 (2)  71 (77)  19 (21) | 0.3227 |
|  | **clinical medical students** | **internship students** | **Physicians** | **P-Value** |
|  | **N= 154 (%)** | **n = 97(%)** | **N= 92 (%)** |  |
| *Demand for the Use of Ultrasound Simulators in Specific Fields* | | | | |
| Use in gynecology  Yes  No  not specified | 88 (57)  37 (24)  29 (19) | 57 (59)  28 (29)  12 (12) | 25 (27)  48 (52)  19 (21) | **<0.001** |
| Use in abdominal ultrasound  Yes  No  not specified | 89 (58)  36 (23)  29 (19) | 76 (78)  9 (10)  12 (12) | 59 (64)  14 (15)  19 (21) | **0.005698** |
| Use in emergency ultrasound  Yes  No  not specified | 87 (56)  38 (25)  29 (19) | 77 (79)  8 (9)  12 (12) | 53 (57)  20 (22)  19 (21) | 0.001245 |
| Use for vascular punctures (CVC)  Yes  No  not specified | 91 (59)  34 (22)  29 (19) | 69 (71)  16 (17)  12 (12) | 40 (43)  33 (36)  19 (21) | **0.001072** |
| Use in intestinal sonography  Yes  No  not specified | 67 (44)  58 (38)  29 (19) | 48 (49)  37 (39)  12 (12) | 38 (41)  35 (38)  19 (21) | 0.8489 |
| Use in echocardiography Yes  No  not specified | 86 (56)  39 (25)  29 (19) | 68 (70)  17 (18)  12 (12) | 52 (57)  21 (23)  19 (21) | 0.1896 |
| Use in other areas  Yes  No  not specified | 1 (1)  124 (80)  29 (19) | 3 (1)  82 (87)  12 (12) | 2 (2)  71 (77)  19 (21) | 0.3682 |
